# Non-Invasive Paleo-Metabolomics and Paleo-Proteomics Analyses Reveal the Complex Funerary Treatment of the Early 18th Dynasty Dignitary NEBIRI (QV30)

**DOI:** 10.3390/molecules27217208

**Published:** 2022-10-25

**Authors:** Elettra Barberis, Marcello Manfredi, Enrico Ferraris, Raffaella Bianucci, Emilio Marengo

**Affiliations:** 1 Department of Translational Medicine, Università del Piemonte Orientale, 28100 Novara, Italy; 2 CAAD, Center for Translational Research and Autoimmune and Allergic Diseases, Università del Piemonte Orientale, 28100 Novara, Italy; 3 Fondazione Museo delle Antichità Egizie di Torino, 10100 Torino, Italy; 4 Dipartimento di Culture e Società, Università di Palermo, 90121 Palermo, Italy; 5 The Ronin Institute, Montclair, NJ 07042, USA; 6 Department of Sciences and Technological Innovation, Università del Piemonte Orientale, 15121 Alessandria, Italy

**Keywords:** non-invasive chemical analyses, paleo-proteomics, paleo-metabolomics, New Kingdom, Nebiri

## Abstract

Biochemical investigations were carried out on the embalmed head of Nebiri (Museo Egizio, Turin; S-5109)—an 18th Dynasty Ancient Egyptian dignitary—and on the canopic jar containing his lungs (Museo Egizio, Turin; S. 5111/02) with the aim of characterizing the organ’s (lung) specific paleo-proteins and of identifying the compounds used in his embalming “recipe”. The application of a functionalized film method allowed us to perform a non-invasive sampling. Paleo-proteomics confirmed the presence of lung tissue-specific proteins (organ specific) as well as the presence of proteins linked to severe inflammation. Paleoproteomics and paleometabolomics further allowed the identification of the main components of Nebiri’s embalming recipe: animal fats and glue, balms, essential oils, aromatic plants, heated Pistacia, and coniferous resins. Both the use of Pistacia and coniferous resins in an early 18th Dynasty individual confirm Nebiri’s high social status. The technique applied offers a targeted approach to the chemical characterization of human tissues, embalming compounds, and organic materials layering in pottery. The ability of the functionalized film method to harvest all types of compounds, from macromolecules (i.e., proteins) to small molecules (i.e., organic acids) opens a new path in the study of ancient material culture; furthermore, it allows to perform untargeted analysis, which is necessary when no a priori information is available.

## 1. Introduction

Over the past two decades, the application of analytical chemistry to bio-archeological materials has made enormous progress. More specifically, through the application of mass spectrometry, minimum amounts of ancient molecules extracted from micro-samples have been identified and quantified [1,2]. Recently, new methods for the non-invasive analysis of cultural materials were developed [3,4,5,6,7,8,9]. Functionalized resins were successfully employed to investigate the compounds used in ancient paintings and frescoes [10,11]; commercially available skin sampling strips were applied to archeological materials to identify paleo-proteins [9]; and other techniques were developed for the identification of in situ hydrogel extraction of proteinaceous binders [5,6] or to reveal the taxonomic identification of ancient archeological materials [8].

Naturally preserved and embalmed bodies from different archeological contexts represent a powerful source of information; over the last decades, investigations of Ancient Egyptian mummies and associated funerary equipment have been extensively performed. For example, Habicht et al. carried out a multidisciplinary investigation on the supposed remains of Queen Nefertari, the royal spouse of pharaoh Ramses II [12]. Similarly, Bianucci et al. performed a multidisciplinary investigation on the embalmed corpses of the Royal Architect Kha and his wife Meryt [13], while Jones et al. identified the embalming recipe and the evolution of early funerary treatments in a prehistoric Egyptian mummy [14]. Proteomics analysis of two 4200-years-old embalmed mummies dated to the First Intermediate Period provided molecular insight into their health conditions, suggesting evidence of acute inflammation and severe immune response [15]. More recently, the application of untargeted metabolomics for chemical characterization of canopic jars’ content and mummy samples from Ancient Egypt led to the identification of thousands of ancient molecules [16,17]; however, no characterization of specific embalming recipes per individual or per organ sampled was obtained [15,16].

The present research focuses on the application of a non-invasive functionalized film method [4] to the analysis of the human remains of an 18th Dynasty Egyptian individual named Nebiri.

## 2. Results and Discussion

### 2.1. Paleo-Proteomic Investigation of Nebiri’s Remains

**Figure 1:**
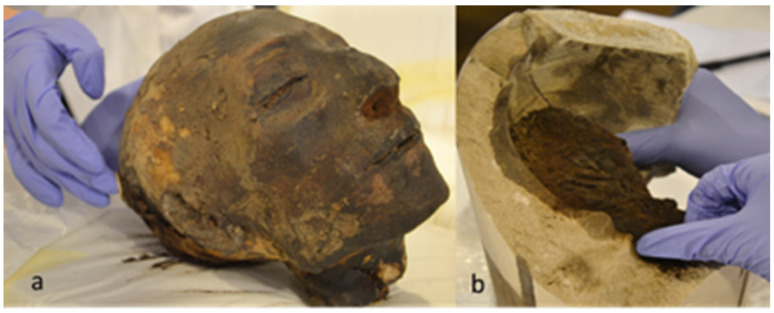
Pictures of the head of Nebiri (**a**) and of the canopic jar containing the lungs (**b**) taken during the non-invasive sampling procedure.

Mass spectrometry was used to sequence ancient protein residues in a dedicated laboratory for the analysis of ancient materials. We applied shotgun proteomics using LC-MS/MS for the analysis of proteins harvested from the head and the lung of Nebiri (Figure 1). The proteins were extracted from the external table of the right parietal bone (in an area where the original textiles were lacking), the scalp, and the lungs using functionalized films; they were then digested prior to the mass spectrometry analysis. Each analyzed sample was preceded and followed by at least one blank injection in order to assess peptide carryover. All the consumables used were new to avoid environmental contaminations. Common protein contaminants were included in the database searches and removed whether identified.

#### 2.1.1. Paleo-Proteins Extracted from the External Surface of the Right Parietal Bone

Several collagens and keratins were identified with the right parietal bone. While human collagen alpha-1(I) chain (CO1A1_HUMAN), collagen alpha-2(I) chain (CO1A2_HUMAN), and collagen alpha-1(III) chain (CO3A1_HUMAN) were the most abundant proteins, two collagens from fowl, namely collagen alpha-1(I) chain (CO1A1_CHICK) and collagen alpha-1(IX) chain (CO9A1_CHICK), with unique and specific peptides, were detected (Table 1). Collagen alpha-2(I) chain (CO1A2_ONCMY) with two unique peptides originated from a fish species was also identified.

As for the presence of collagens from fowl, the proteins CO1A1_CHICK and CO9A1_CHICK from *Gallus gallus* were identified. Both proteins were characterized by ancient modifications, indicating that the collagen was a component of the original material and did not come from contamination with more recent conservation treatments. While it is reported that animal glues were widely used as binders and adhesives, especially in Egyptian cartonnage, here the first scientific evidence of a proteinaceous material—an animal glue from fowl—directly extracted from the surface of a human ancient bone is provided.

Interestingly, domestic fowls were raised during the 18th Dynasty as reported in the literature [18]. In addition, the presence of fish collagen suggests that fish glue adhesives were also used. Although Ancient Egyptian records do not describe their preparation process in detail, it is known that these adhesives were used in the embalming procedures. Fishing was a highly diffused practice in Ancient Egypt, and fish glue would have been made by melting fish/fish scraps over a fire and then applied with the help of a brush/or a spatula [19].

The fish collagen identified has been classified as belonging to the species *Oncorhynchus mykiss* (Rainbow trout) and not to the Nile Tilapia, as one would have expected; this result is mostly likely due to the fact that the full protein database of Tilapia spp. is not yet available and only a few proteins have been characterized thus far. Although the use of fish oil was reported in a previous study on Egyptian mummies [13], the authors did not find specific markers attributed to this kind of animal oil.

#### 2.1.2. Lung Proteins

**Figure 2:**
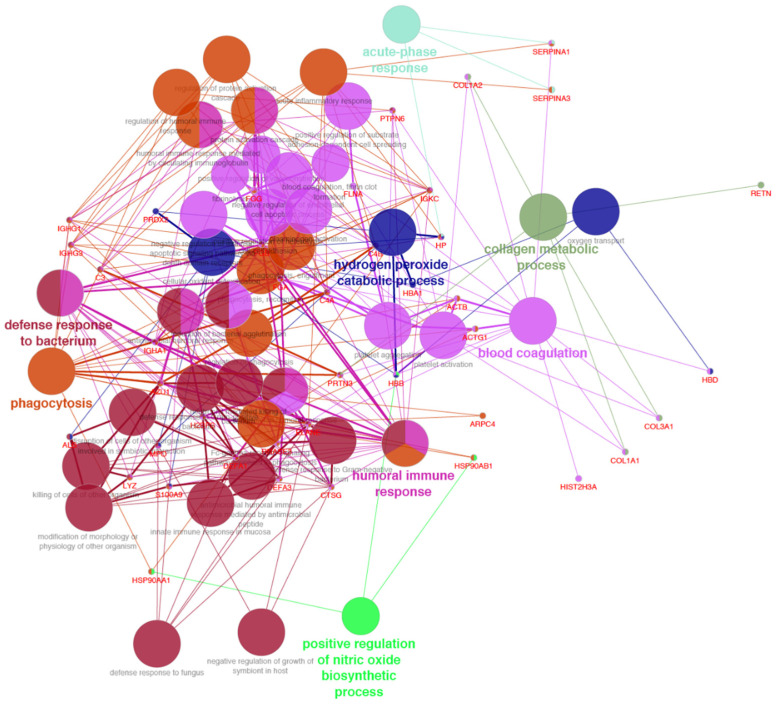
Cytoscape-based ClueGo pathway analysis and visualization. Enriched pathways were obtained from the Kyoto Encyclopaedia of Genes and Genome (KEGG) database. The figure reports the functionally grouped networks of identified proteins. Terms are linked based on κ-score (≥0.4); edge thickness indicates the association strength while node size corresponds to the statistical significance for each term. Biological processes are also reported.

The proteomic analysis of lung samples revealed the presence of 60 unique human proteins (Table 1); among them are several specific biomarkers of lung tissue. Lung is undoubtedly a major “immunological organ” since it contains a considerable amount of lymphoid tissue. We identified neutrophil defensins (DEF1_HUMAN), which are antimicrobial peptides present in large amounts in the neutrophil [20]; hemoglobins (HBA_HUMAN and HBB_HUMAN), which are involved in oxygen transport from the lung to the various peripheral tissues; the neutrophil serine proteases cathepsin G (CATG_HUMAN) and neutrophil elastase (ELNE_HUMAN), which are involved in immune-regulatory processes and exert antibacterial activity against various pathogens [21]; and haptoglobin (HPT_HUMAN), which is known to be associated with the host-defense response to infection and inflammation and is expressed at a high level in lung cells [22]. The identified proteins were also subjected to gene ontology classification based on biological processes with the Cytoscape software and the ClueGO plug-in. The analysis showed several lung biological processes such as immune response, defense response to bacteria, and oxygen transport (Figure 2). No proteins of animal origin were detected.

#### 2.1.3. Proteins from the Scalp

Proteins extracted from the scalp were mainly human collagens (CO1A1_HUMAN, CO1A2_HUMAN and CO3A1_HUMAN) and human keratins (K1C9_HUMAN and K1C10_HUMAN). Besides these proteins, an animal collagen, namely collagen alpha-2(I) chain (CO1A2_CHICK), was identified. All these proteins bear modifications specific for ancient materials; in addition, the presence of collagen from fowl confirmed the use of an original treatment with an ancient animal glue.

#### 2.1.4. Ancient Protein Damage

Although proteins survive longer than DNA, they still decay naturally over time. The identification of diagenetic protein modifications was used to distinguish paleo-proteins from modern ones. Hydroxylation of proline, which is one of the main modifications of collagen, was identified in all analyzed samples. Deamidation and aminoadipic acid from lysine are more specifically related to degradation. Deamidation is usually associated with protein biological aging; it plays an important role in protein degradation and has been correlated with the time of aging [23]. Aminoadipic acid from lysine is the most important age-dependent form of oxidative damage. The presence of 2-aminoadipic acid in the ancient samples can be associated with the decomposition that occurred immediately after Nebiri’s death [24]. As reported in Table 2, all samples taken from the parietal bone, lung, and head skin were characterized by the presence of deamidation and aminoadipic acid from lysine. These results confirmed the ancient origin of the samples analyzed.

### 2.2. Identification of Small Molecules with Untargeted Metabolomics Analysis

Paleo-metabolomic analysis was performed to investigate small molecules extracted from Nebiri’s lungs, from the textiles, which originally wrapped the lung, and from the inner surface of the lung’s canopic jar; this was done in order to identify the chemical composition of the embalming “recipe” used to treat the body of the ancient Egyptian dignitary. The paleo-molecules were extracted using functionalized films and then analyzed using mono-dimensional and comprehensive GCxGC-MS. The complete list of identified molecules across the samples are reported in Supplementary Table S2.

#### 2.2.1. Textiles Wrapping the Lungs

The main components characterized in the lung’s textile samples were fatty acids, which originally derive from triglycerides. Their distribution indicates that the major compounds are myristic, palmitic, and stearic acids together with few amounts of margaric, oleic, arachidic, azelaic, pimelic, and sebacic acids. The presence of cholesterol and of odd-chain-length fatty acids combined with the presence of squalene, an isoprenoid hydrocarbon abundant in skin lipids, clearly indicates the presence of animal fats [25,26]. Furthermore, the presence of oleic acid as the only unsaturated fatty acid and the absence of nonadecylic acid indicates that the origin of animal fatty acids may be from non-ruminant animals (porcine or fowl) [27]. All these samples were collected from the external side of the bandages, a condition which highly minimizes the possibility of contamination from the lung.

Moreover, the lipid profile points to the use of a plant oil or a mixture of plant oils. In fact, the presence of azelaic, pimelic, and sebacic acids, which form by the degradative oxidation of originally unsaturated fatty acids, suggests the presence of a vegetable oil [28]. The presence of plant oil impregnating the funerary textiles was already observed as the main balm component in previous studies on pharaonic embalming agents [14,27,28,29].

Aromatic acids characteristic of plant products, vanillic acid, benzoic acid, 4-hydroxybenzoic acid, 4-methoxyphenol, and 4-methylbenzoic acid, were also present in the textiles. These compounds, together with vegetable tannins identified in the samples (pyrogallol, hydroquinone, and myo-inositol), confirm the use of plant extracts although it is impossible to determine the vegetal genus/species. The presence of sucrose and D-mannopyranose suggests the use of a plant gum or sugar as a component of the balm; again, it is not possible to establish the original source, i.e., Acacia.

Coniferous resin was identified in the lung’s bandages samples. The mass spectra signal of 15-Hydroxy-7-oxodehydroabietic acid, typical of aged conifer resin, was present along with the terpenoid borneol; however, since borneol can derive from many different plant extracts/essential oils or resins, it cannot be considered a marker of specific botanical species. Beeswax markers and proteinaceous materials, such as animal glue, egg, or milk, were not present in the analyzed samples.

#### 2.2.2. The Jar Containing the Lung

The analysis of the samples taken from the inner surface of the jar containing the lung revealed a slightly different chemical composition. The main fatty acids identified were palmitic and stearic acids, with few amounts of myristic acids and traces of margaric, azelaic, and pimelic acids. The palmitic/stearic ratio of two and the presence of azelaic and pimelic acids indicate the use of a plant oil together with animal fats, as confirmed by the identification in traces of the odd-chain fatty acids pentadecanoic and margaric acids.

The inner surface of the jar was also covered by traces of vegetable tannins such as pyrogallol, hydroquinone, and 4-methylcatechol, thus confirming the use of plant extracts; the conifer resin biomarker 15-Hydroxy-7-oxodehydroabietic acid was also identified.

#### 2.2.3. Lung

The chemical composition of the lung samples were more complex compared with those taken from the textiles and the jar samples. The hydrolysis and oxidation of lung triglycerides and glycolipids generated a different distribution of monocarboxylic and dicarboxylic acids. Palmitic and stearic acids are the main components, followed by myristic, azelaic, and pimelic acids, while margaric and arachidonic are present only in traces.

The presence of odd-chain-length fatty acids and of three cholesterol degradation products, namely 3,7-bis[(trimethylsilyl) oxy]cholest-5-ene, (3ß,7ß)-, 7-ketocholesterol, and cholesta-3,5-dien-7-one confirmed that the analyzed sample is human pulmonary tissue, as already shown by previous histological investigations [30]. The metabolomics analysis also identified other metabolites, especially sugars, formed during the hydrolysis of glycolipids.

The oxidized 15-Hydroxy-7-oxodehydroabietic acid characteristic of the coniferous resin was identified together with D-Pinitol and Resorcinol. Vegetable tannins such as Scyllo-Inositol, o-Toluic acid, Pyroglutamic acid, 3-Phenyllactic acid, 2-furoic acid, 4’-Hydroxyacetophenone, and Myo-Inositol confirmed the use of plant extracts.

Although the most specific triterpenoid of masticadienonate series (moronic, masticadienoic, isomasticadienoic, and oleanonic acids) have not been detected in the monodimensional GC-MS analysis, in the bidimensional GCxGC analysis, we found other biomarker compounds, generally used to identify an antique Pistacia resin (Supplementary Table S1) [31]: penta and tetra-cyclic triterpenes from resins of Pistacia species as oleanane-type molecules, in particular β-Amyrin, with most abundant fragment ion at *m/z* 218 and Olean-18-en-3-ol, o-TMS with main fragment ions at *m/z* 189 and 204; and a triterpenoid with dammarane skeleton, Dammaran-3-one, 20,24-epoxy-25-hydroxy with characteristic base peak at *m/z* 143 [32]. In this case, as well-described by Daifas et al. [33], we confirmed that Pistacia resins were used for embalming treatments, probably for their antibacterial, antifungal, and antiseptic properties [34] or they might have been employed in the preparation of kyphi ointment, as well as used for its religious significance [35]. Although various authors reported the presence of two species of Pistacia, *P. lentiscus* and *P. atlantica* [36], we were not able to discriminate the different species within the sample, possibly due to the very low abundance of characteristic markers. However, the presence of Pistacia and other triterpenoid compounds lead us to confirm that the embalming “recipe” used for Nebiri was expensive and typically reserved for the royalty or for extremely wealthy nobles and notables [37].

Finally, vegetable tannins and phenols with antiseptic activities, derived from plants as catechol and guaiacol, have been found. The presence of guaiacol in ancient recipes might be linked to the use of wood smoke/cedar wood in the embalming process [38]. The GCxGC-MS analysis also allowed the identification of juniperol (Cupressaceae family), used as an embalming substance in mummies and canopes [16] (Table 3).

## 3. Materials and Methods

### 3.1. Nebiri’s Lung and Head

Nebiri was an ancient Egyptian dignitary who lived 3500 years ago under the reign of Thutmose III (1479–1424 BCE; 18th Dynasty). His tomb (QV30) was discovered in the Valley of the Queens, in 1904, by the first director of the Egyptian Museum of Turin, Ernesto Schiaparelli (1856–1928). Unfortunately, his tomb was plundered in antiquity by grave robbers and his mummy was deliberately destroyed. Only his head (S.5109) and the canopic chest (S. 5110; s. 5111/02; S-5112; S-5113) were preserved. Previous studies showed that the man described as “Chief of Stables” died of acute decompensation of chronic heart failure when he was 45–60 years old [39].

In the present study, non-invasive sampling was carried out on Nebiri’s head and on the canopic jar containing his lung (S. 5111/02). Access to the content of this specific canopic jar was granted by Museo Egizio of Turin (Italy) since the apical portion of the vase was already broken and portions of the lungs and linen textiles were easily accessible. Paleo-proteins, small molecules, and lipids were identified without damaging the mummy’s head and his funerary vase whose long-term preservation is required by the current legislation on Cultural Heritage (Figure 1a,b). Therefore, non-invasive sampling was performed on Nebiri’s scalp, on the canopic jar containing the lung, on the lung tissue, and on the textiles originally used to wrap both the head and organ. The functionalized film was placed in contact with the surface of each sample for 5 min, and then removed leaving the original ancient materials unmodified.

### 3.2. Small Molecules Extraction and Derivatization

Small molecules were extracted from the surface of the samples using three different functionalized films: (i) a mix-bed cation/anion exchange film, (ii) a C4 resin film, and (iii) a C8 resin film. Briefly, the films are based on ethyl-vinyl acetate (EVA) as a binder of ground AG 501 mix-bed cation/anion exchange (from Biorad, Hercules, CA, USA), of C4 resin and of C8 resin (both from Sigma, Darmstadt, Germany), respectively. The mixture of melted EVA and resins were extruded in the form of a thin film in laboratory a week before the use. Prior to their use on the object, the functionalized films were humidified with ultrapure water and then the water was discharged. The films were positioned with extreme caution on the surface of the samples for 10 min. The metabolite extracts were then eluted from the film with 1 mL of ethanol for 30 min. Then, the strips were removed and the metabolites were subjected to derivatization. The derivatization protocol was performed by adding 20 µL of methoxamine hydrochloride in pyridine (20 mg/mL) and 30 µL of BSTFA. Samples were incubated at 80 °C for 20 min after every addition, and then centrifuged for 15 min (14,500× *g* at RT). Nitrogen steam was used to gently dry the samples before the gas-chromatography analysis.

### 3.3. Proteins Extraction and Digestion

The proteins were extracted from the surface of the samples using a film functionalized with a mix-bed cation/anion exchange and C8 resins, as previously described by Barberis et al. [3,11]. The functionalized film was humidified with ultrapure water and then the water was discharged. The film was positioned with extreme caution on the surface of the sample for 10 min. The protein extracts were then eluted from the film with 500 μL of 1.0 M ammonium acetate in a tube for 30 min. Then, the strip was removed and the proteins were first denatured with TFE at 60 °C, reduced with 200.0 mM DTT, alkylated with 200.0 mM IAM, and finally digested with trypsin overnight. The peptide digested were desalted on the Discovery^®^ DSC-18 solid phase extraction (SPE) 96-well Plate 25 mg/well (Sigma-Aldrich Inc., St. Louis, MO, USA).

### 3.4. GC-MS and GCXGC-MS Analyses

Gas chromatography–time of flight mass spectrometry (GC-TOF/MS) was performed using an Agilent 7890B GC (Agilent Technologies, USA) and Pegasus (BT) TOF-MS system (Leco Corporation, USA) equipped with an Rxi–5 ms column (30 m × 0.25 mm × 0.25 μm, RESTEK, USA), stationary phase 5% diphenyl-95% dimethyl polysiloxane. High-purity helium (99.999%) was used as the carrier gas at a flow rate of 1.00 mL/min^−1^. Samples were injected in splitless mode at 280 °C. The chromatographic conditions were as follows: initial temperature 40 °C, 5 min isothermal, 8 °C/min up to 300 °C, 20 min isothermal. The MS parameters were as follows: electron impact ionization source temperature (EI, 70 eV) was set at 250 °C; scan range 40/630 *m/z*, with an extraction frequency of 30 kHz. The chromatograms were acquired in TIC (total ion current) mode. Mass spectral assignment was perfomed by matching with NIST MS Search 2.2. Libraries, implemented with the MoNa Fiehns Libraries. For the 2D analysis, a LECO Pegasus BT 4D GCXGC/TOFMS instrument (Leco Corp., St. Josef, MI, USA) equipped with a LECO dual stage quad jet thermal modulator was used. The GC part of the instrument was an Agilent 7890 gas chromatograph (Agilent Technologies, Palo Alto, CA, USA), equipped with a split/splitless injector. The first-dimension column was at 30 m Rxi-5 ms capillary column (Restek Corp., Bellefonte, PA, USA) with an internal diameter of 0.25 mm and a stationary phase film thickness of 0.25 μm, and the second-dimension chromatographic column was a 2 m Rxi-17Sil MS (Restek Corp., Bellefonte, PA, USA) with a diameter of 0.25 mm and a film thickness of 0.25 μm. The carrier gas (helium) was used with a flow rate of 1.4 mL/min. The secondary column was maintained at +5 °C relative to the GC oven temperature of the first column. Additionally, the MS method was the same as the mono-dimensional analysis, while the extraction frequency was 32 kHz, the acquisition rates was 200 spectra/s, and the modulation period was maintained at 4 s for the entire run. The modulator temperature offset was set at +15 °C relative to the secondary oven temperature, while the transfer line was set at 280 °C [40,41].

### 3.5. LC-MS Analysis and Data Processing

The extracted proteins were analyzed with a micro-LC Eksigent Technologies system (Eksigent, Dublin, OH, USA) that included a micro LC200 Eksigent pump with flow module 5–50 μL, interfaced with a 5600+ TripleTOF system (AB Sciex, Vaughan, ON, Canada) equipped with DuoSpray Ion Source and CDS (Calibrant Delivery System). The stationary phase was a Halo C18 column (0.5 × 100 mm, 2.7 μm; Eksigent Technologies Dublin, USA). The mass spectrometry data were searched using Mascot (Mascot v. 2.4, Matrix Science Inc., Boston, USA) and Protein Pilot; the digestion enzyme was trypsin, with 1 missed cleavage. The instrument was set to ESI-QUAD-TOF and the following modifications were specified for the search: carbamidomethyl (C) as fixed modification, Acetyl (K), Deamidated (NQ), Gln->pyro-Glu (N-term Q), Glu->pyro-Glu (N-term E), Hydroxylation (KP), Lys-> AminoadipicAcid (K), Oxidation (M), Oxidation (P), Trp->Kynurenin (W) as variable modification and hydroxylation of prolines and lysines when collagen was present [25]. A search tolerance of 0.1 Da was specified for the peptide mass tolerance, and 50 ppm for the MS/MS tolerance. The charges of the peptides to search for were set to 2+, 3+, and 4+, and the search was set on monoisotopic mass. The databases employed were Swissprot human reviewed (version 11032016, containing 42,179 sequence entries), cRAP (proteins commonly found in proteomics experiments that are present either by accident or through unavoidable contamination), and Metazoa (version 10082018, containing 103,419 sequences). Only peptides with individual ion scores > 20 were considered for identification purposes. Only proteins presenting two or more unique peptides, after screening for possible contaminants, were considered positively identified.

## 4. Conclusions

Previous studies pointed to the fact that Nebiri was a high social status individual. A virtopsy performed on the undamaged head of Nebiri showed an extremely careful cosmetic treatment. A similar treatment was observed only in Yuya and Tjuiu, the parents of Queen Tye, the royal spouse of King Amenhotep III (1388–1348 BCE). Nebiri’s high status could also be inferred by the title “Chief of Stables”; at the beginning of the 18th dynasty, “ownership of horses and their stables were frequently reserved for high-ranking officials and those related to royalty” [30,38]. The results of the paleo-proteomics and paleo-metabolomics investigation confirm all previous findings. Nebiri’s embalming “recipe” was composed of a mixture of animal fat and glue, balms, essential oils, aromatic plants, heated Pistacia, and coniferous resins. Pistacia and coniferous resins, non-native imported resins from the north-eastern Mediterranean, were considered luxury goods and only available for royal and high elite consumption [30]. Although quantitative information related to the single components of the recipe is very hard to obtain on these ancient and complex materials, our results suggest the dominance of plant oil and animal glue in funerary textiles, which are the major “balm” component, while aromatic plant extracts and gums were added to the mixture in minor amounts. Finally, conifer resins and Pistacia were identified only in trace amounts and can be considered the minor components of the embalming recipe.

From a chemical point of view, the method we used in this research offers an appropriate and non-invasive approach for the identification and characterization of human, vegetable, and animal paleo-proteins and paleo-metabolites in ancient remains. The ability of the film to harvest all types of compounds, from macromolecules such as proteins, to small molecules like organic acids, is fundamental to study ancient remains as well as fragile objects; furthermore, it allows performing an untargeted analysis, which is necessary when no a priori information is available. Lastly, the method is very rapid, thus allowing high sample throughput; the adsorbed molecules can be analyzed with any kind of analytical instrument in dedicated settings.

## Figures and Tables

**Table 1:** Proteins identified in the head and in the lung of Nebiri. Sample, proteins, accession name, score, and number of peptides identified for each protein are reported.

Sample	Proteins	Accession Name	Score	N. of Peptides
External table of the right parietal bone	Collagen alpha-1(I) chain	CO1A1_HUMAN	1436	23
	Collagen alpha-2(I) chain	CO1A2_HUMAN	1127	22
	Collagen alpha-1(I) chain	CO1A1_CHICK	764	12
	Collagen alpha-2(I) chain	CO1A2_ONCMY	66	3
	Keratin, type I cytoskeletal 9	K1C9_HUMAN	464	5
	Keratin, type I cytoskeletal 10	K1C10_HUMAN	236	5
	Keratin, type II cytoskeletal 1	K2C1_HUMAN	225	5
	Keratin, type II cytoskeletal 2 epidermal	K22E_HUMAN	40	3
	Keratin, type II cytoskeletal 5	K2C5_HUMAN	39	3
	Collagen alpha-1(IX) chain	CO9A1_CHICK	207	3
	Collagen alpha-1(III) chain	CO3A1_HUMAN	82	2
Lung tissue (most abundant proteins)	Serum albumin	ALBU_HUMAN	942	12
	Protein S100-A9	S10A9_HUMAN	640	6
	Ig alpha-1 chain C region	IGHA1_HUMAN	469	5
	Alpha-1-antitrypsin	A1AT_HUMAN	376	7
	Cathepsin G	CATG_HUMAN	367	5
	Hemoglobin subunit beta	HBB_HUMAN	350	3
	Histone H2A type 1	H2A1_HUMAN	340	3
	Isoform H14 of Myeloperoxidase	PERM_HUMAN	288	7
	Alpha-1-antichymotrypsin	AACT_HUMAN	236	6
	Collagen alpha-1(III) chain	CO3A1_HUMAN	210	5
	Ig gamma-1 chain C region	IGHG1_HUMAN	209	5
	Hemoglobin subunit alpha	HBA_HUMAN	190	3
	Neutrophil defensin 1	DEF1_HUMAN	177	3
	Lysozyme C	LYSC_HUMAN	166	2
	Histone H4	H4_HUMAN	158	2
	Histone H2B type F-S	H2BFS_HUMAN	137	4
	Peroxiredoxin-2	PRDX2_HUMAN	125	2
	Actin, cytoplasmic 1	ACTB_HUMAN	120	2
	Tubulin beta-2B chain	TBB2B_HUMAN	118	4
	Fibrinogen beta chain	FIBB_HUMAN	110	4
	Neutrophil elastase	ELNE_HUMAN	104	2
	Isoform Gamma-A of Fibrinogen gamma chain	FIBG_HUMAN	100	3
	Band 3 anion transport protein	B3AT_HUMAN	77	2
	Isoform 2 of Haptoglobin	HPT_HUMAN	77	2
	Isoform 2 of Complement C4-A	CO4A_HUMAN	76	3
	Isoform 2 of Heat shock protein HSP 90-alpha	HS90A_HUMAN	76	2
	Myeloblastin	PRTN3_HUMAN	75	2
Scalp	Collagen alpha-1(I) chain	CO1A1_HUMAN	1145	12
	Collagen alpha-2(I) chain	CO1A2_HUMAN	1010	13
	Collagen alpha-2(I) chain	CO1A2_CHICK	134	3
	Collagen alpha-1(III) chain	CO3A1_HUMAN	400	9
	Keratin, type I cytoskeletal 9	K1C9_HUMAN	327	5
	Keratin, type I cytoskeletal 10	K1C10_HUMAN	103	3

**Table 2:** Ancient modifications identified in proteins extracted from Nebiri’s remains. The name of the protein, type of modification, and number of modified amino acids in each sample for each protein are reported.

Protein	Modification	Scalp	Parietal Bone	Lung
Collagen alpha-1(I) chain	Deamidated (NQ)	8	11	4
Collagen alpha-1(III) chain	Deamidated (NQ)	3	2	2
Collagen alpha-2(I) chain	Deamidated (NQ)	3	4	1
	Lys-> AminoadipicAcid (K)	-	1	-
Collagen alpha-5(VI) chain	Deamidated (NQ)	-	1	-
Collagen alpha-3(VI) chain	Deamidated (NQ)	-	-	1
Collagen alpha-1(IX) chain	Deamidated (NQ)	1	-	-
Collagen alpha-1(V) chain	Deamidated (NQ)	-	-	1
	Lys-> AminoadipicAcid (K)	-	-	1
Collagen alpha-1(XXVIII) chain	Deamidated (NQ)	-	1	-
Collagen alpha-1(X) chain	Lys-> AminoadipicAcid (K)	-	1	-

**Table 3:** Nebiri’s embalming “recipe”: list of main class of compounds associated with the samples and relative assignment.

Class of Compound	Sample	Recipe Products
Linear monocarboxylic saturated fatty acids; Dicarboxylic acids; Hydroxycarboxylic acids; Monounsaturated fatty acids	Lung, canopic jar, head	Plant oils and relative oxidation products
Diterpenoids	Lung, canopic jar, head	Pinaceae resins
Aromatic acids	Lung, canopic jar, head	Vegetable balms
Monosaccharides	Lung, canopic jar, head	Human tissue or gums
Triterpenoids	Lung	Pistacia resin
Tannins	Lung, canopic jar, head	Cedar oil/wood smoke
Collagen proteins	Scalp	Animal glue (fish and fowl)

## Data Availability

Not applicable.

## Supplementary Materials

The following supporting information can be downloaded at: https://www.mdpi.com/article/10.3390/molecules27217208/s1, Table S1: Complete list of identified molecules. Table S2: Heated Pistacia markers obtained from the lung sample with the untargeted analysis by GCxGC-MS; the name of the molecules, formula, retention time in first and second dimension and similarity > 700 (index of identification reliability) are given.
