# Supplementary material for: Non-Invasive Paleo-Metabolomics and Paleo-Proteomics Analyses Reveal the Complex Funerary Treatment of the Early 18th Dynasty Dignitary NEBIRI (QV30)
Source: Molecules. 2022 Oct 25;27(21):7208. doi: 10.3390/molecules27217208 (PMC9653799; doi:10.3390/molecules27217208)
Supplement: Supplementary file 1 [file molecules-27-07208-s001.zip › Supplementary table 2 nebiri.pdf]

Table S2: Heated *Pistacia* markers obtained from the lung sample with the untargeted analysis by GCxGC-MS; the name of the molecules, formula, retention time in first and second dimension and similarity > 700 (index of identification reliability) are given.

**GCxGC-MS**			
Name	***Formula***	***R.T. (s)***	***Similarity***
S28-Norolean-17-en-3-one 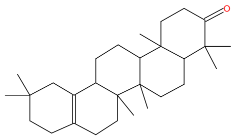 m/z 163.16	$C_{29}H_{46}O$	2474,88 (1st)<br>2,840 (2nd)	790
Olean-18-en-3-ol, O-TMS, (3β) 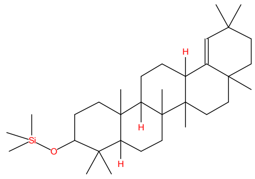 m/z 189.17	$C_{33}H_{58}OSi$	2484,88 (1st)<br>2,616 (2nd)	877
Β-Amyrin 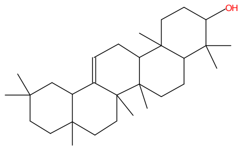 m/z 218.21	$C_{30}H_{50}O$	2479,88 (1st)<br>3,846 (2nd)	779
Dammaran-3-one,20,24-epoxy-25hydroxy 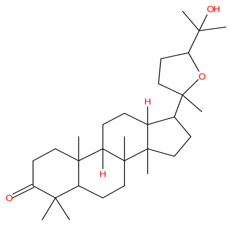 m/z 143.11	$C_{30}H_{50}O_3$	2639,87 (1st)<br>1,014 (2nd)	797
